# “Pain is not typically taken into consideration due to him being nonverbal”- emergency department experiences among persons with disabilities: a mixed methods study in Kingston, Ontario

**DOI:** 10.3389/fresc.2024.1353120

**Published:** 2024-07-25

**Authors:** Minha Haque, Sierra Gaspari, Nicole Bobbette, Melanie Walker, Susan A. Bartels

**Affiliations:** ^1^ Department of Emergency Medicine, Queen’s University, Kingston, ON, Canada; ^2^ Department of Public Health Sciences, Queen’s University, Kingston, ON, Canada; ^3^ Department of Biomedical and Molecular Sciences, Queen’s University, Kingston, ON, Canada; ^4^ School of Rehabilitation Therapy, Queen’s University, Kingston, ON, Canada

**Keywords:** equity-deserving groups, emergency department, intellectual disability, persons with disabilities, physical disability, sensory disability, substance use

## Abstract

**Background:**

Persons with disabilities (PWD) are more likely to visit the emergency department (ED) and often have complex health needs when accessing care in the ED. Yet there is limited understanding of ED care experiences among PWD, especially in a Canadian context. The aim of this study was to examine the ED care experiences of PWD in contrast to a comparison group in Kingston, Ontario to better understand their health care needs.

**Methods:**

A mixed-methods study with a community-based participatory approach examining participants’ past ED care experiences (within 24 months) was conducted in Kingston, ON. Quantitative data from those with disabilities and those from the comparison group were compared using chi squared tests to identify differences between groups. An inductive and deductive thematic analysis approach was used to identify themes in the shared qualitative data. Convergence of findings across quantitative and qualitative data was undertaken.

**Results:**

A total of 175 participants identified as having a disability. In contrast with the comparison group (*N* = 949), PWD were more likely to report being given too little attention to their needs (*p* < 0.001), that it was more important to be treated with kindness/respect than to receive the best possible medical care (*p* < 0.001), to report feelings of disrespect and/or judgement (*p* < 0.001), and that better understanding of personal identity/situation/culture and better communication would improve ED care. Qualitative analysis highlighted the following themes: poor communication between PWD and health care providers (HCP), compassionate medical care received, perceived HCP negative attitudes/beliefs related to having a disability and substance misuse, and perceived HCP lack of knowledge/skill to treat the unique health needs of PWD.

**Conclusion:**

Findings highlight the need to improve ED care for PWD. Future quality improvement initiatives should focus on incorporating a deeper understanding of disability into medical education and emergency medicine (EM) residency education, designing curricula that emphasize cultural humility, and implementing community-based placements providing opportunities for health professionals to work with and learn from PWD.

## Background

In Canada, one in five people aged 15 years and over are living with some form of disability, equating to approximately 6.2 million Canadians (1). Despite this significant presence, research examining the experiences of persons with disabilities (PWD) in emergency department (ED) remains limited (2–4). The existing research primarily focuses on health care provider (HCP) or personal caregiver perspectives and consists mainly of smaller qualitative studies. These studies often do not include different disability subgroups, nor do they incorporate a comparison group to evaluate whether experiences among those with disabilities are unique (3–6).

Research examining HCP and caregiver perspectives suggests that health inequalities faced by PWD stem from systemic, political, and societal factors rather than the disabilities themselves (7). Discriminatory attitudes and behaviors among HCPs, coupled with inadequate training, contribute to health disparities for PWD, leading to care avoidance and worse health outcomes (8, 9). Inadequate access to healthcare can result in a higher risk of premature mortality (10). Diagnostic overshadowing, where the presence of a disability overshadows unrelated health concerns, leads to delayed diagnoses and inadequate treatment for acute health issues (11). This issue is compounded by insufficient knowledge about specific disabilities among HCPs. For example, nurses in emergency units in Ireland have reported heavy reliance on caregivers when treating patients with intellectual or developmental disabilities (IDD) due to inadequate training and understanding, posing ethical dilemmas (12). Recognizing the unique needs of PWD is essential for addressing historical discrimination and improving ED care experiences (11).

Some research on patient perspectives and potential suggestions to improve the healthcare system has been published in the United States, United Kingdom, and Australia. However, these findings may not directly translate to Canada due to differences in the healthcare system (8, 13–15). For instance, Canada's public healthcare system provides essentially “free” medical care, leading to increased demand and longer wait times for specialist appointments and high-tech scans compared to private healthcare systems (16). In contrast, the United States offers Medicare as a uniformed national public health insurance for aged and disabled individuals, further distinguishing the ED experiences of Canadian vs. American PWD (16). This research from other countries underscores the need for tailored approaches to address the unique challenges faced by PWD in Canadian EDs, considering the distinct healthcare system and its impact on access and care quality.

## Introduction

PWD encompass a wide range of physical and cognitive health conditions, with varying experiences based on their specific health conditions and capacities. PWD often have complex health needs and face unique challenges in accessing healthcare services (2, 8, 17, 18). EDs plays a crucial role as a primary gateway for acute care and connecting patients to necessary community supports (8). However, the ED environment can be intimidating and overwhelming, particularly for PWD whose needs may not be adequately understood or accommodated by HCPs (3, 8).

The Canadian health care system is in a crisis which directly impacts EDs across the country (19). At times there have been urgent calls for HCP to step into nursing roles to keep EDs running, EDs have closed in rural areas due to lack of staff, and there is an ongoing shortage of family physicians, subsequently increasing ED visits (19, 20). Following the COVID-19 pandemic, patients and HCP alike expressed that overcrowding and long wait times in the ED impact the quality of care that is provided and received (21, 22). EDs have been described as a complex, overwhelming, and stressful environment by many and this feeling is only exacerbated by equity-deserving groups (EDG), specifically PWD (18, 22). PWD are one of many equity-deserving groups that often experiences discrimination within the healthcare system. According to the Government of Canada, an equity-deserving group is defined as a group of individuals who, because of systemic discrimination, face barriers that prevent them from having access to the same resources and opportunities as other members of society (23). Attitudinal, social, historical, and environmental challenges based on economic status, race, age, ethnicity, disability, gender, and/or sexual orientation create inequities for EDG in our society (7). Given these challenges, understanding the healthcare disparities faced by PWD in the ED becomes imperative.

The healthcare disparities experienced by PWD reflect the broader oppressive narratives and actions that permeate society. The stigma PWD face increases the likelihood of receiving inadequate medical care and decreases the probability of having their needs properly addressed (7). Negative ED care experiences can lead to care avoidance and deteriorating health outcomes (24). There is a pressing need to better understand the ED experiences of PWD to identify and address ongoing issues. Out of concern for these health inequities, we conducted a mixed-methods study with a community-based participatory approach to answer the following research question: What are the ED care experiences of adults with a disability in Kingston, Ontario?

## Methods

### Study design

A mixed-methods, cross-sectional study was conducted with a community-based participatory approach (25). This study is a secondary data analysis focusing on PWD in contrast to a comparison group Drawing on community-based participatory research principles, community partners helped throughout the project to inform survey questions, collect and analyze data and disseminate results.

### Setting

Kingston is a city located in southeastern Ontario, Canada with a population of approximately 132,000 people (26). The population is comprised of a mix of different ethnicities, with a significant proportion of people of European descent. The median age of the population is forty-one, reflecting a balanced population of young people, working-age residents, and retirees. Kingston's economy is driven by the public sector, including institutions such as Queen's University, St. Lawrence, College, Royal Military College, and Kingston Health Sciences Centre consisting of the Kingston General Hospital (KGH) and Hotel Dieu Hospital (HDH) (27).

The data were collected at the KGH ED and HDH urgent care centre (UCC) from June to August 2021 by trained research assistants (RAs) from Monday to Friday between 9am to 9pm. To capture participants who were not actively seeking care in the ED/UCC, due to previous negative experiences, RAs also surveyed clients visiting community partners including Ongwanada Resource Centre, Kingston Health Sciences Centre's Vision Rehabilitation Clinic, Independent Living Centre Kingston, and Providence Care Hospital-Mental Health and Rehabilitation. All surveys were completed in the ED, UCC, or at a community partner's office with no further follow-up.

### Participants & recruitment

Between June to August 2021, any individual aged 16 and older, medically stable, proficient in English, and entering the ED, UCC, or the office of community partners during study hours were invited to participate in the study. Participants either self-identified or were identified by a personal caregiver as having an intellectual, sensory, physical disability, and/or neurodivergence. For the purposes of this study, we used a comparison group that was comprised of individuals who did not identify as being a member of any EDG. The reasons provided by participants who were approached but declined to participate were also documented. Participants were also given the opportunity to share more than one experience and thus complete more than one survey each. Participants were asked to indicate how many times they had completed the survey, and the number of initial submissions was considered as the total number of unique participants.

## Data collection

### Survey instrument tool

Spryng.io is a sensemaking narrative capture tool that extracts meaning from micronarratives shared by participants on a topic of interest (28). Participants were prompted to audio-record micronarratives in response to an open-ended question regarding an ED care experience within the preceding 24 months (see Appendix 1 for questions), thereby generating the qualitative data After briefly sharing a *prior* ED care experience, participants interpreted these experiences through a set of predesigned questions by plotting their perspectives between two possible options (sliders) or three options (triads) (see Appendix 1 for examples). These responses were then quantified by the Spryng.io program generating the quantitative data. Multiple-choice questions (MCQs) collected demographic data and other information about the ED visit to help contextualize the micronarrative. The survey, delivered in English, took fifteen minutes to complete and all data for this study were collected using the Spryng.io narrative capture tool on handheld tablets. As this work is an extension of a broader parent study, the MCQs also gave participants the opportunity to self-identify with up to three equity-deserving groups (EDGs) that were most relevant to their ED care experience. However, for this paper, the focus is specifically on PWD and thus the analysis and discussion will not encompass the other EDGs. Those results will be published separately. The survey was created by the research team in conjunction with community partners. The community partners also facilitated connections with PWD who were able to provide input on the survey questions.

#### Primary outcome

The primary outcome was self-reported ED care experiences among PWD in comparison with those who do not identify as equity-deserving.

### Data analysis

#### Quantitative analysis

Using SPSS (IBM SPSS Statistics V.26.0.0.0), descriptive statistics were calculated using chi squared tests to examine differences between participants who self-identified as having a disability compared with those who did *not* identify as equity deserving (comparison group). *P*-values less than 0.05 were considered statistically significant. Spryng.io data were exported to Tableau (V.2022.2) where collective plots were visually analysed to identify patterns (clusters, extremes, and outliers) in the data. The slider and triad data were then disaggregated based on self-identification as a PWD or participants that did not identify as equity-deserving (comparison group). Slider data were analyzed as histograms with the collective areas under the bars for each group determined using the Kruskal-Wallis H test and chi-squared tests in SPSS (IBM SPSS Statistics V.26.0.0.0) to determine if the bar areas were statistically different between groups. Violin plots show the overall distributions of responses for the slider questions, with an asterisk indicating the overall mean for each group. For triads, geometric means for each group were calculated using R Scripts (R V.3.4.0), along with 95% confidence intervals (CI), which are presented graphically as 95% confidence ellipses. If two 95% confidence ellipses did not overlap, it was concluded that the corresponding geometric means were statistically different. A list of the dyad and triad questions can be found in Appendix 1.

#### Qualitative analysis

Micronarratives shared by participants who self-identified as PWD were independently coded both deductively and inductively by two team members (MH and SG) using NVivo for Mac v12.7.0 (29). An initial codebook was developed based on existing literature and the sensemaking survey, but codes were also added inductively from the micronarratives themselves. Both team members independently coded the entire dataset with the first 20 micronarratives being coded together to gauge and establish a consistent approach and improve inter-coder reliability. The remaining micronarratives were coded independently with periodic comparison to maintain consistency and resolve discrepancies. A thematic analysis was then completed to identify themes, allowing the research team to establish connections between the research objective and the findings. Themes were supported by cross-referencing exact quotations from the micronarratives and participants’ own interpretation through the quantitative sensemaking data.

#### Mixed-methods analysis

In this study, triangulation of the quantitative and qualitative data was done. This approach strengthens the robustness of findings by mitigating the limitations and biases inherent in individual methods. Once response patterns were identified through the quantitative analysis, the themes identified during the qualitative analysis were reviewed to facilitate the interpretation of the statistical findings. Examples of quotes are included to illustrate the mixed-methods findings.

#### Engagement with local community

Focus group discussion (FGD) participants were recruited through community partners. The aim was to discuss the study findings with community members, specifically to gather their perspectives on the data and whether the results resonated with their own knowledge and previous ED experience in Kingston. The focus group also provided an opportunity for PWD and their caregivers to share further care experiences and suggest improvements for ED care for PWD in the futurOne FGD, lasting two hours, was conducted with community partners and PWD to share study findings. This FGD was held at Ongwanada, a facility that supports persons with developmental disabilities, with a focus on those with complex needs and their families (30). The FGD facilitated by an expert in disabilities (NB), included four PWD along with their caregivers and was crucial for validating the study findings and ensuring that the lived experiences of PWD were accurately represented. The questions used to solicit discussion can be found in Appendix 2.

With permission from attendees, the FGD was audio recorded and subsequently transcribed for detailed analysis. The data from the focus group discussions were analyzed using a thematic grouping approach, structured around the predefined questions used during the discussions. After transcription, the notes were carefully reviewed and organized according to these established themes, such as participants’ reactions to the study findings and suggested strategies for improving ED experiences. This thematic organization allowed for a targeted and systematic analysis of the participants’ responses, ensuring the data was aligned with the research objectives.

#### Ethical considerations

All study participants provided informed consent by tapping a box on the survey in the handheld tablet. All data were anonymous from the point of collection since no identifying information was collected. The research team provided a $5 coffee gift card to each sensemaking survey participant as a token of appreciation. Focus group participants provided verbal consent and were each provided with a $10 coffee gift card and refreshments. The Queen's University Health Sciences and Affiliated Teaching Hospitals Research Ethics Board approved this study protocol (#6029400).

## Results

### Characteristics of study participants

In total, 4,414 potential participants were approached with 2,579 declining to take part in the study. 1,973 unique participants participated and shared 2,114 experiences about their ED care experiences. This included 949 who did *not* identify as equity-deserving (comparison group) and 994 who identified as equity-deserving. For this study objective, 184 surveys were provided across 175 unique PWD or personal caregivers.

Demographic and ED visit characteristics are provided in Table 1. PWD were more likely to report being aged 65 or older (*p* = 0.0002) and were more likely to identify as being White/European (*p* < 0.0001). Additionally, PWD were more likely to struggle to meet their needs (i.e., food, housing, clothing) (*p* < 0.0001), were more likely to have a greater frequency of ED visits within the last two years (*p* < 0.0001) and to indicate that they felt disrespected and/or judged while in the ED (*p* < 0.0001).

**Table 1:** Participant demographics and ED visit characteristics disaggregated by self-identification as having a disability vs. not identifying as equity-deserving (comparison group).

Variable	Total (*N* = 1,133)	Persons with a disability	Comparison group	*P*-value*
		(*n* = 184)	(*n* = 949)	
	*n* (%)	*n* (%)	*n* (%)	
Age				**0**.**0,002**
<18	100 (9.0)	7 (4.0)	93 (10.0)	
18–25	114 (10.0)	21 (11.0)	277 (29.0)	
26–45	205 (18.0)	38 (21.0)	167 (18.0)	
46–65	224 (20.0)	58 (32.0)	166 (17.0)	
>65	213 (19.0)	60 (33.0)	153 (16.0)	
No data	277 (24.0)	0 (0.0)	277 (29.0)	
Gender identity				0.08
Woman	628 (55.0)	111 (60.0)	517 (54.0)	
Man	479 (42.0)	68 (37.0)	411 (43.0)	
Non-binary	8 (1.0)	3 (2.0)	5 (1.0)	
No data	18 (2.0)	2 (1.0)	16 (2.0)	
Identify as gender diverse				0.26
Yes	6 (1.0)	2 (1.0)	4 (0.0)	
No	1,066 (94.0)	174 (95.0)	892 (94.0)	
No data	61 (5.0)	8 (4.0)	53 (6.0)	
Sexual orientation of patient				0.06
Asexual	5 (0.0)	2 (1.0)	3 (0.0)	
Bisexual	38 (3.0)	11 (6.0)	27 (3.0)	
Gay/lesbian	13 (1.0)	2 (1.0)	11 (1.0)	
Pansexual	12 (1.0)	3 (2.0)	9 (1.0)	
Straight	976 (86.0)	146 (79.0)	830 (87.0)	
Questioning/unsure	4 (0.0)	2 (1.0)	2 (0.0)	
Sexual orientation not on this list	2 (0.0)	0 (0.0)	2 (0.0)	
No data	83 (7.0)	18 (10.0)	65 (7.0)	
Frequency of “struggling to make ends meet” (SES)				**<0**.**0001**
Never	595 (53.0)	71 (39.0)	524 (55.0)	
Rarely	181 (16.0)	26 (14.0)	155 (16.0)	
Sometimes	154 (14.0)	23 (13.0)	131 (14.0)	
Often	24 (13.0)	24 (13.0)	34 (4.0)	
All the time	54 (5.0)	22 (12.0)	32 (3.0)	
No data	91 (8.0)	18 (10.0)	73 (8.0)	
Ethnic identity				**<0**.**0001**
White/European	746 (66.0)	156 (85.0)	590 (62.0)	
Indigenous	22 (2.0)	13 (7.0)	9 (1.0)	
Black	5 (0.0)	1 (1.0)	4 (0.0)	
Other (Latin American, South	42 (4.0)	3 (1.0)	39 (4.0)	
Asian, Southeast Asian, West Asian, other)				
One or more ethnicity	11 (1.1)	4 (2.0)	7 (1.0)	
No data	307 (27.0)	7 (4.0)	300 (32.0)	
ED visit frequency in last 2 years prior				**<0**.**0001**
0	219 (19.0)	33 (18.0)	185 (20.0)	
1–3	403 (36.0)	68 (37.0)	335 (35.0)	
≥ 4 times	101 (10.2)	54 (18.0)	47 (6.9)	
No data	30 (16.0)	30 (16.0)	365 (38.0)	
Focus of experience				0.20
Healthcare providers (doctors, nursing staff)	513 (45.0)	112 (61.0)	401 (42.0)	
Other hospital staff (social workers, security officers, porters, and imaging technicians)	26 (2.0)	10 (5.0)	16 (2.0)	
Waiting room	24 (13.0)	24 (13.0)	88 (9.0)	
Other hospital areas (triage, registration, discharge)	73 (6.0)	17 (9.0)	56 (6.0)	
Other (not specified)	44 (4.0)	6 (3.0)	38 (4.0)	
No data	365 (32.0)	15 (8.0)	350 (37.0)	
Effect of identity, personal situation, and/or culture on experience				**<0**.**0001**
In a very good way	36 (3.0)	7 (4.0)	29 (3.0)	
In a good way	58 (5.0)	8 (4.0)	50 (5.0)	
In a very bad way	17 (2.0)	13 (7.0)	4 (0.0)	
In a bad way	38 (3.0)	20 (11.0)	18 (2.0)	
No effect	837 (74.0)	122 (66.0)	715 (75.0)	
No data	147 (13.0)	14 (8.0)	133 (14.0)	
Experience was about being treated without respect				**<0**.**0001**
Yes	122 (11.0)	37 (20.0)	85 (9.0)	
No	922 (81.0)	135 (73.0)	787 (83.0)	
No data	89 (8.0)	12 (7.30)	77 (8.0)	
Overall feelings about ED experience				**0**.**0003**
Positive	633 (56.0)	83 (45.0)	550 (58.0)	
Mixed	104 (9.0)	25 (14.0)	79 (8.0)	
Negative	311 (27.0)	69 (38.0)	242 (26.0)	
No data	85 (8.0)	7 (4.0)	78 (8.0)	
Identification with more than 1 EDG				**<0**.**0001**
0	949 (84.0)	0 (0.0)	949 (100.0)	
1	103 (9.0)	103 (56.0)	0 (0.0)	
2	42 (4.0)	42 (23.0)	0 (0.0)	
3	39 (3.0)	39 (21.0)	0 (0.0)	

Bold values are statistically significant values.

Variables were collapsed from original survey response options due to small cell sizes and to improve clarity.

* Chi-squared tests were used and did not include missing data/”not sure/prefer not to say”.

Table 2 outlines the self-identified disability types included for analysis. The study population was defined by those who self-identified as having a disability and then identified as having one of: hearing loss/deafness, low vision/blindness, intellectual disabilities, physical disabilities, or neurodivergence (i.e., learning disabilities, autism spectrum disorder).

**Table 2:** Disability types included in the study.

Type of disability	Total
	(*N* = 184)
	*n* (%)
Hearing loss/deafness	11 (6.0)
Low vision/blindness	5 (3.0)
Intellectual disability	17 (9.0)
Neurodivergence (learning disability, autism spectrum disorder)	29 (16.0)
Physical disability	122 (66.0)

## Mixed-methods findings

### Poor communication between PWD and HCPs

#### Quantitative results

**Figure 1:**
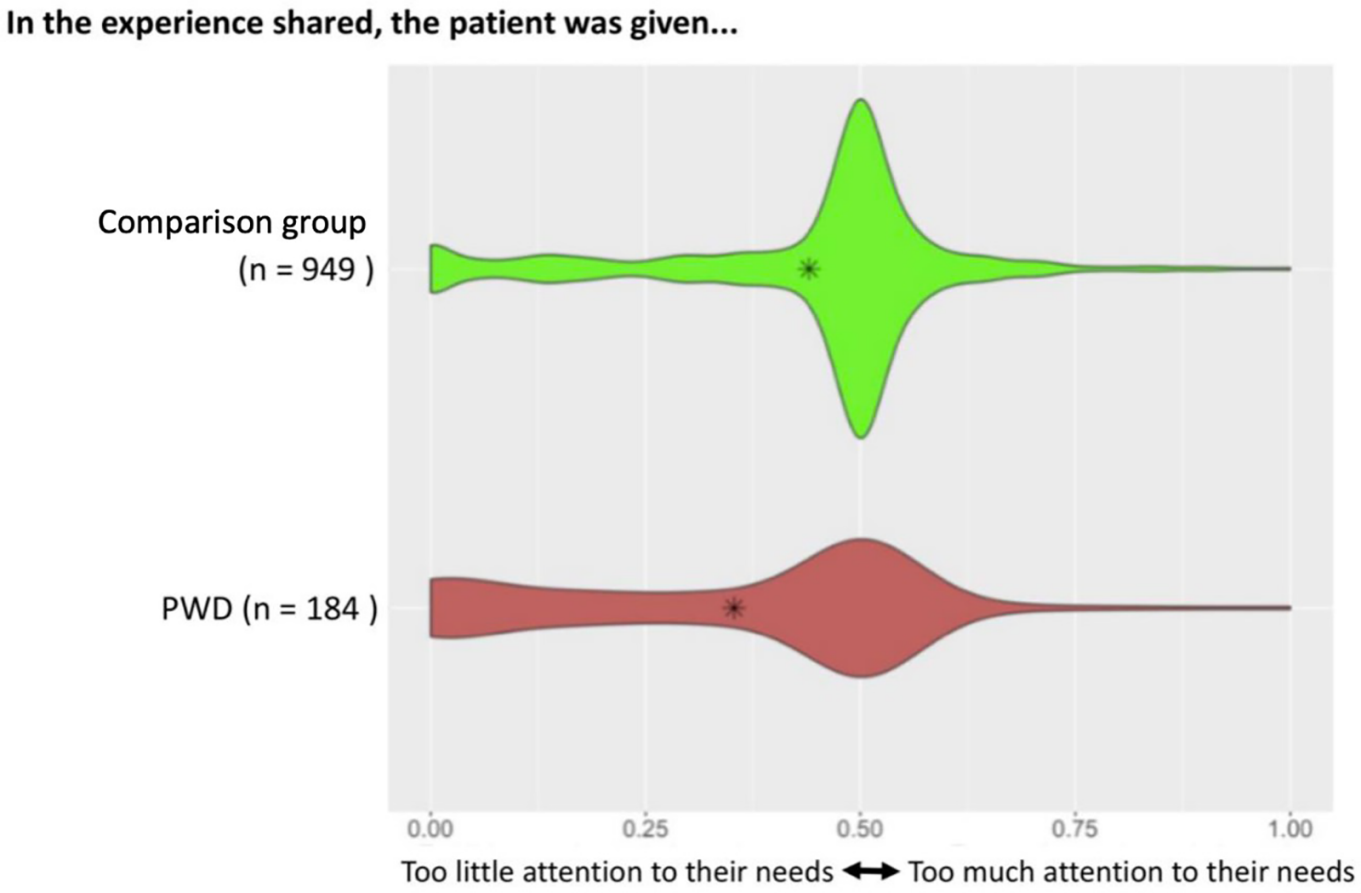
Slider asking participants about attention given to their needs. Plot of responses along the spectrum of “too little attention to their needs” vs. “too much attention to their needs” disaggregated by whether the patient identified as a PWD or as part of the comparison group. Asterisks indicate the overall mean for each group.

Figure 1 provides an example of a slider that asked about the attention given to a patient's needs. The shape of each violin plot for the PWD and the comparison groups illustrate the distribution of participant's responses. PWD were *more likely* to report that their needs were given too little attention as shown by the wider base to the left (*p* < 0.001).

#### Qualitative results

Participants shared ED experiences about poor communication between the patient and ED staff, which strongly contributed to the perception of needs not being met. PWD often reported that ED staff did not listen to their concerns, did not provide clear discharge instructions, and/or did not involve them in decisions related to their medical care.

Two individuals who identified as PWD, shared the following:

*“My experience in hospital has not been very pleasant. I believe that healthcare professionals need to work together as a team rather than always just focusing on one issue with the patient. And I think that most people know themselves very well…I think it is important that healthcare providers listen to the patient or the patient*’*s friends or family members who know them best.”*

- Person with sensory disability (low vision/blindness)

*“I came with my daughter and her son (my grandson). The doctor said to come back if he got worse and he did so we came back. But then his nose started bleeding, so we came back by ambulance 3 or 4 times before they admitted him. He has autism. I felt insulted when the doctor didn*’*t want to give him blood tests and really listen to his symptoms.”*

- Family member of a person with Autism Spectrum Disorder

### Compassionate care

#### Quantitative results

**Figure 2:**
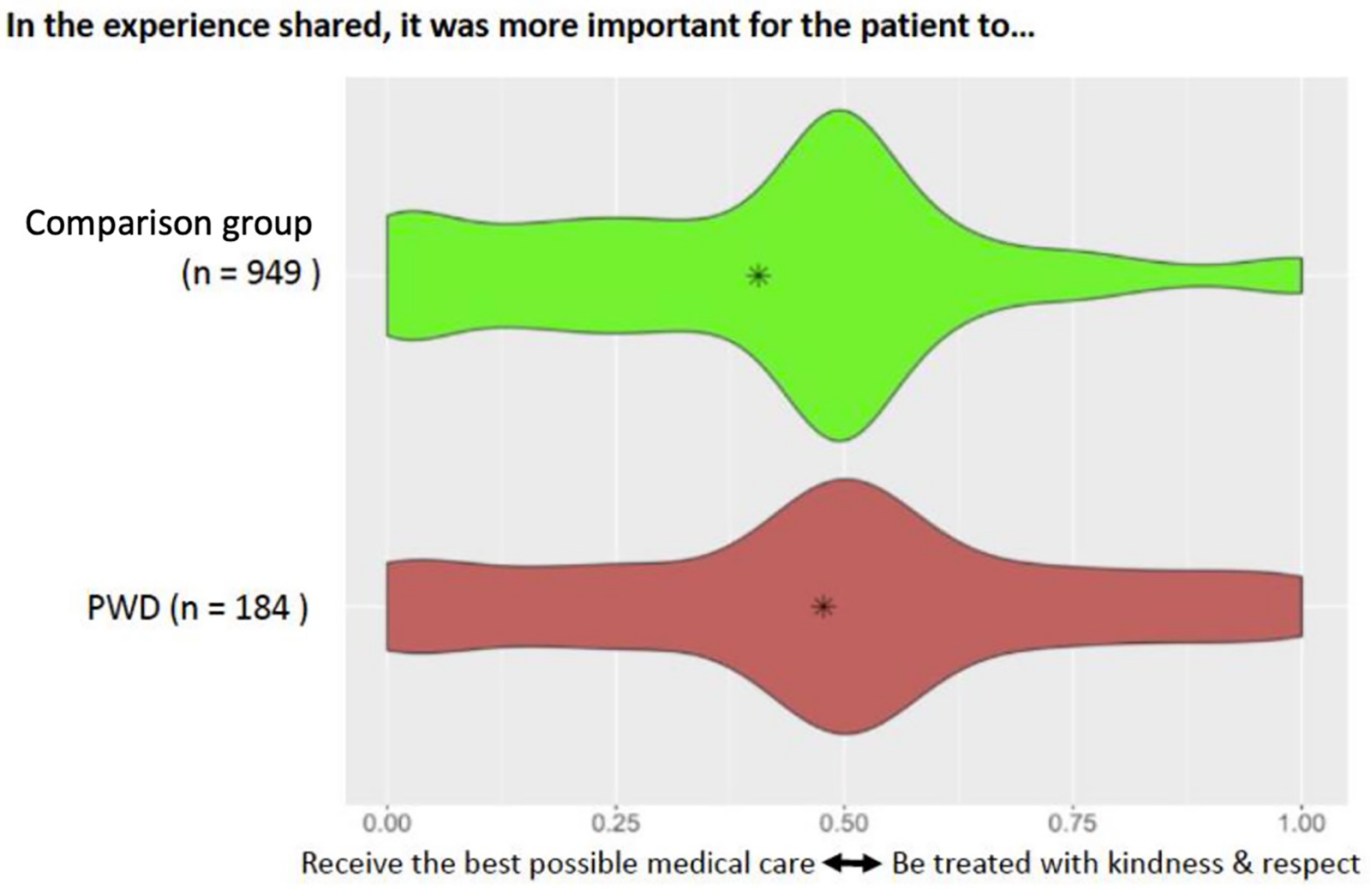
Slider asking participants about the importance of receiving good medical care compared to being treated with kindness and respect. Plot of responses along the spectrum of “receive the best possible medical care” vs. “be treated with kindness and respect” disaggregated by whether the patient identified as a PWD or as part of the comparison group. Asterisks indicate the overall mean for each group.

In contrast to the comparison group, PWD were more likely to report that it was more important to be treated with kindness and respect than to receive the best possible medical care (*p* < 0.001) (Figure 2).

#### Qualitative results

PWD felt that HCPs provided compassionate care during their visit, reporting that staff took initiative to treat their health concerns and went “beyond the call of duty”, contributing to a satisfactory ED experience.

A PWD and caregiver of a PWD shared the following:

*“I found that the triage nurse to be, um, very efficient but friendly and pleasant. And even though I had some trouble hearing because I was having hearing difficulty, she was most willing to repeat the questions and make things clear. And then it was not a very long wait in the waiting room. And then I got right in, and I was looked after. And then there was a fair wait for the doctor. The nursing staff were most accommodating. And the student nurse who came to do get the blood was very, very friendly and really interested in what she was doing, and her overseer was helpful to her but wasn*’*t overpowering to her. The doctor was tremendous.”*

- Person with hearing loss/deafness

*“My daughter was suffering from multiple seizures. At the time she had about 40 of them. We were unable to get them to stop. So off we went to the emergency. And she was attended by X at the time. And the positive experience that came out of that was of course, immediately they were able to stop the seizures…all in all the care that she received was above and beyond. Stopping the multiple seizures that she was having was a very positive experience for us. And we still benefit from them to this day.”*

- Mother of a child with an intellectual disability

### HCP negative attitudes/beliefs related to having a disability and/or substance misuse

#### Quantitative results

**Figure 3:**
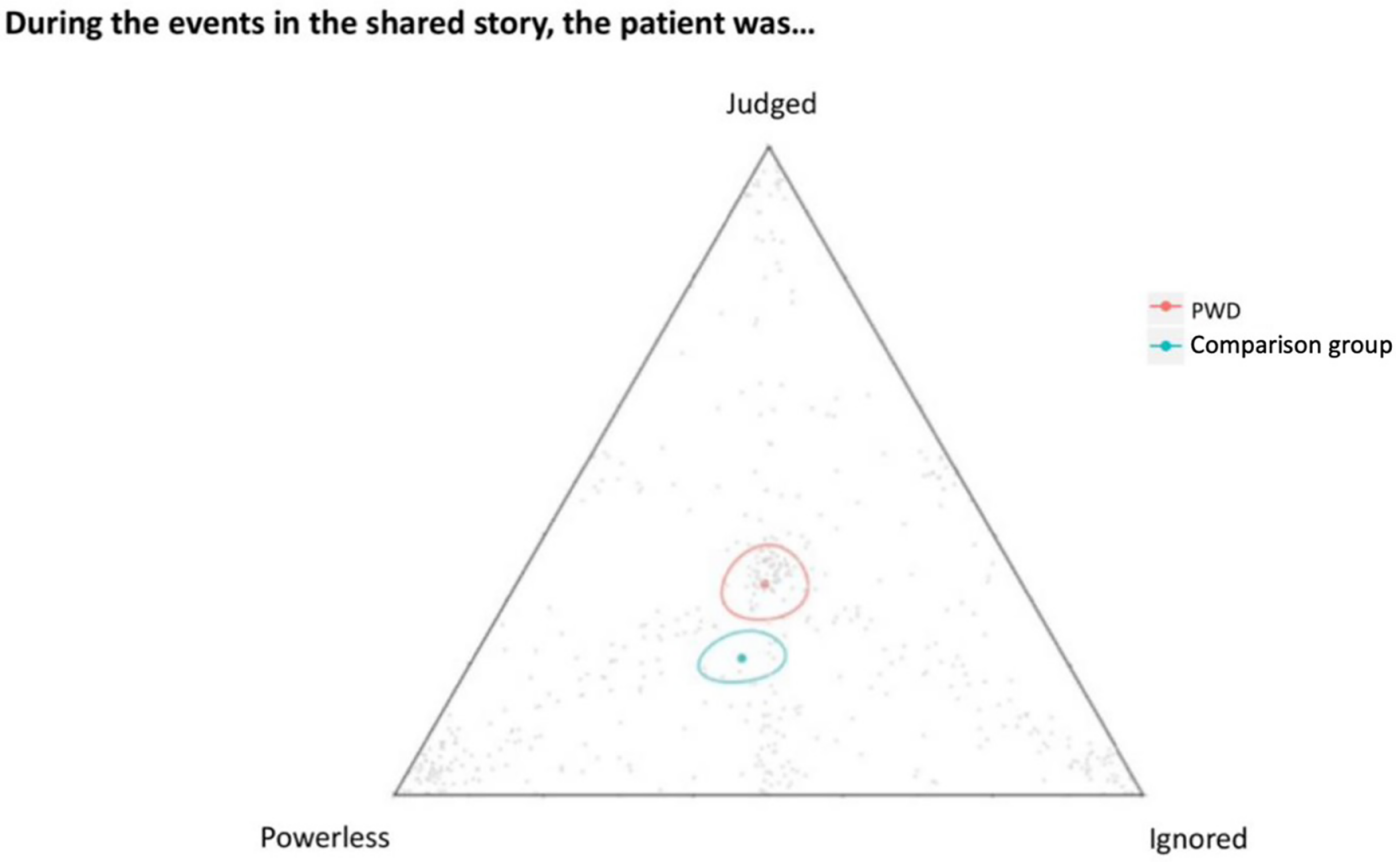
Each individual grey dot represents a participant's response. The red and green dots represent geometric means for PWD and the comparisongroup, respectively and are each surrounded by a 95% CI.

The triad shown in Figure 3 asked patients to interpret their feelings about the events shared in the micronarrative (i.e., judged, powerless/not in control, and ignored). PWD were statistically *more likely* to report feelings of judgement while receiving care in the ED as evidenced by the 95% confidence ellipses that do not overlap.

#### Qualitative results

PWD felt that HCPs held *negative feelings and beliefs* related to having a disability. Additionally, PWD often reported that HCPs inaccurately labelled them as substance users and felt repeatedly dismissed when they provided an explanation. This caused patients to feel judged, ignored, and powerless while receiving ED care.

Two individuals who identified as PWD, shared the following:

*“I went to the ER by ambulance for excessive bleeding from my period (dysmenorrhea), and although I explained that I already took pain medication, I felt like the nurses didn*’*t believe me (or possibly thought I was lying and on drugs)…Once again, while in the most intense pain ever, I had nurses asking me if I’m sure I didn*’*t take any illegal drugs. It wasn*’*t until the ER doctor came over and realized the pain was being caused by bloating and gave me medication to aid gas pain. Within 15 min my symptoms subsided, and I was able to walk out of the ER.”*

- Person who identifies as neurodivergent

*“I went in to KGH for psych. I have 2 types of autism. I was very suicidal. And also agitated. I can*’*t sit still and I was very scared. The lady there accused me of being on crystal meth. She asked me if I’ve ever done meth. I told her I’ve never done any drugs. She acted like I was lying to her. She also read my chart to all the other nurses and they were laughing at me and making fun of me. Nobody believed me when I told the doctor about it.”*

- Person with Autism Spectrum Disorder

### Perceived HCP lack of knowledge/skill to treat unique health needs of PWD

#### Quantitative findings

**Figure 4:**
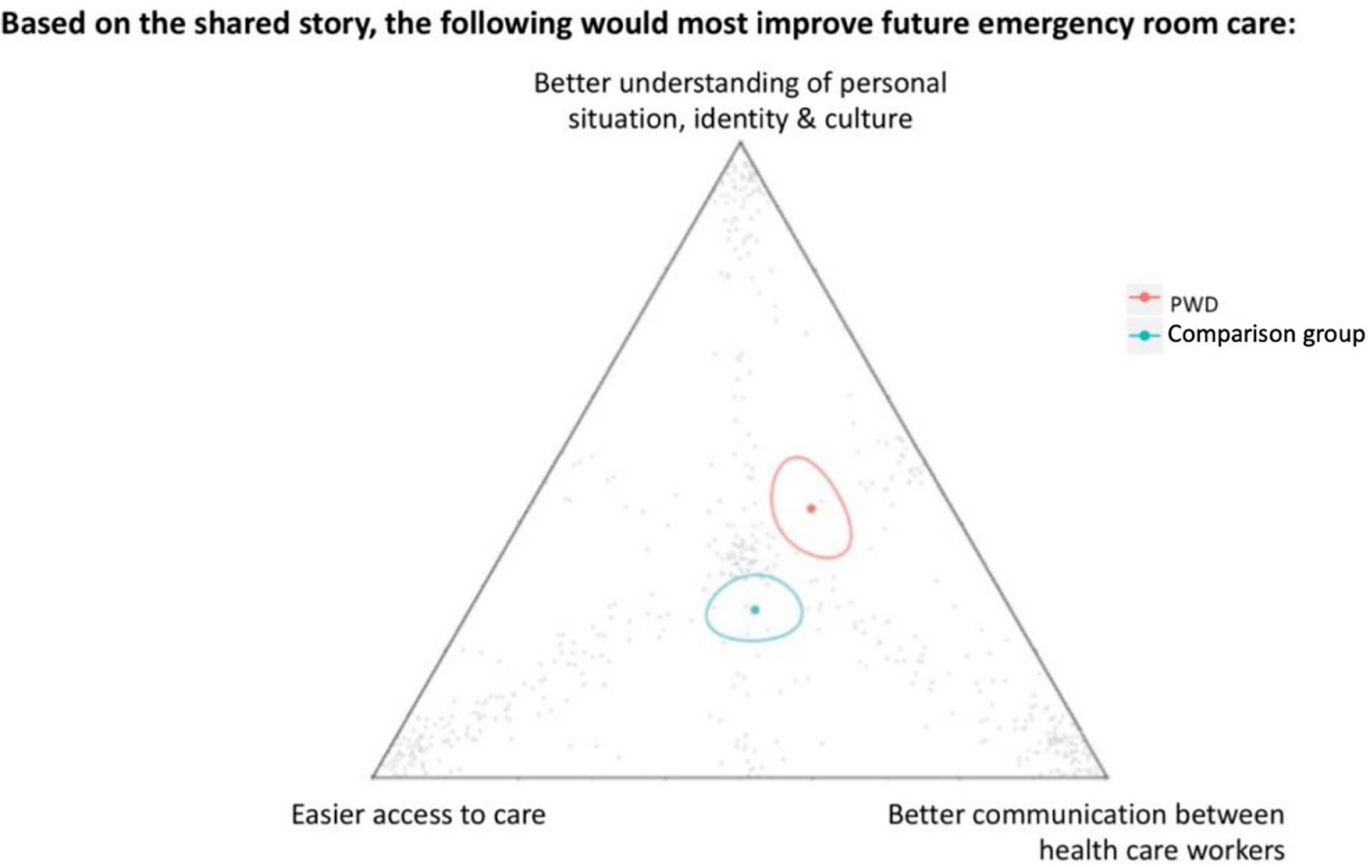
The individual grey dots represent a participant's response. The red and green dots represent geometric means for PWD and the comparison group, respectively and are each surrounded by a 95% confidence ellipse.

PWD were asked what would most improve future ED care, with possible options of better understanding of their personal situation, identity, and culture, easier access to medical care, and better communication between health care workers (Figure 4). PWD were significantly *more likely* to indicate that a better understanding of their personal situation, identity and culture was needed.

#### Qualitative results

PWD reported that *HCP lacked the knowledge and skills to competently treat many of their health concerns* and which may contribute to the feelings among PWD that a better understanding of personal situation/identify/culture is needed*.* Additionally, participants often reported that there was a lack of communication between HCPs. This caused patients to be moved frequently between departments and have repeat visits to the ED.

A PWD and caregiver of a PWD shared the following:

*“I accompanied a client to KGH where he was often forgotten about due to his loud behaviour. When it came to diagnosis, they would say that they weren*’*t going to do certain tests/studies due to his difficult behaviours and cooperation. We were also lied to about with his diagnosis. One resident informed us that he did not have a bowel obstruction but when we received discharge paperwork it clearly stated he did and that was what he was treated for. Often, we are given band aid fixes to this client*’*s medical issues instead of looking for the route cause. This client is tossed around between different departments at every visit he has, typically they do short term fixes, and he returns back within 6 months with the same issues. This client*’*s pain is also not typically taken into consideration due to him being nonverbal.”*

- Caregiver of a person with an intellectual disability

*“…I was shoveled from an ambulance stretcher into the waiting room which bothered me immensely because I was immobile. I didn*’*t have means to get around. And they just stuck me in a wheelchair, so I was at the mercy of the security guard to help me in the waiting room…What bothered me more than anything was the lack of accessibility for someone my size. I couldn*’*t get out of hospital beds or structures because they are too high. I couldn*’*t use commodes because they are too high for somebody my size. Hospital gowns were far, far too large. And there was a fear of tripping. So, I was relegated to the bed.”*

- Person with physical disability

### Additional findings

The qualitative data identified positive medical care as an important theme among participants. PWD reported receiving exceptional medical care including diagnoses, testing, and symptom treatment.

A PWD and caregiver of a PWD shared the following:

*“…They did a bunch of x-rays on me and blood tests. They were all so kind. I was amazed at how good the doctor was. He knew exactly what was wrong and got to my test results promptly…”*

*-* Person who identifies as neurodivergent

“*A physician saw her and ruled out a blood clot within a very few minutes by skillfully manipulating her leg. He was most impressive with respect to his skill and his demeanor toward a ninety-five-year-old.”*

- Family member of a person with a sensory disability (hearing loss/deafness)

### Focus group discussions

FGDs were conducted with community partners and PWD to share study findings and to solicit their input on how best to improve ED care. When asked about poor communication between PWD and HCP, participants with hearing impairments emphasized that communication could be improved if HCP spoke loudly, maintained eye contact with the PWD, and clearly laid out instructions for next steps.

When asked about receiving compassionate and positive medical care, FGD participants spoke of how kind ED staff were. One participant highlighted that the staff made the effort to call their family members who were not present in the ED and inform them of the treatment plan and next steps. This action reinforced the idea of the staff “going beyond the call of duty” for the participant.

With respect to negative attitudes/beliefs related to having a disability, substance misuse, and/or a perception that HCP lacked knowledge/skills to meet the unique health needs of PWD, FGD participants highlighted the importance of caregivers accompanying them to the ED. PWD emphasized that having their caregivers present would not only make them more comfortable in the ED but would also improve communication between all individuals involved in providing care.

When asked about improvements to the ED, FGD participants stated that they would like more independence and they dislike when staff try to do everything for them. Participants stated they would like the opportunity to try things themselves and will ask for assistance, if necessary.

## Discussion

Study findings suggest that PWD were more likely to report negative ED care experiences and felt judged and disrespected by HCPs. Quantitative results highlight that PWD were more likely to report being given too little attention to their needs when visiting the ED, and this was partially attributed to poor communication between patients and HCP. Specifically, PWD reported that ED staff did not listen to their concerns, did not provide clear discharge instructions, or involve them in decisions pertaining to their medical care. This finding was consistent with existing research which has shown that communication difficulties between HCPs and PWD pose a significant barrier in accessing quality health care (31–34). Hemsley et al. suggested that EDs are fast paced, stressful environments, with intense time constraints. With limited time, HCPs may find it difficult to adapt their communication approach to fit the needs of PWD and are more likely to rely on caregivers to communicate on behalf of the patient (31). This highlights the need for more HCP training around treating the specific health needs of PWD and how to adjust their approach to meet the needs of those with disabilities.

Our findings also suggest that PWD were more likely to report that being treated with kindness/respect was more important than receiving the best possible medical care. Accompanying micronarratives highlighted that PWD reported that they received compassionate care from HCPs. Specifically, PWD described HCPs took the initiative to treat their health concerns and went above and beyond regarding their treatment. Going beyond “the call of duty” has been identified as an enhancer to appropriate hospital care for PWD, however reports of this in the literature are limited (35).

PWD were more likely to report feeling judged in the ED in contrast to the comparison group, which is consistent with the findings from previous literature (3, 34, 36, 37). PWD attributed these negative feelings to attitudes and beliefs held by HCPs related to having a disability. This finding has been shown extensively in earlier research. For example, Lewis and Stenfert-Kroese found that staff reported negative attitudes and emotions in caring for PWD, impacting the quality of care received (32). It is noteworthy that our study identified new insights into PWD being inaccurately labelled as substance users. Certain disability groups have been shown to suffer disproportionately from substance misuse (38). However, based on the findings by Chapman and Wu, the prevalence of illicit drug use, particularly in intellectual disabilities, is low (39). Some PWD may display challenging behaviours in the ED, especially in environments not conducive to their needs. This, coupled with lack of experience and sufficient training, might lead HCPs to attribute their behaviour to substance use. More research is warranted into the negative attitudes of HCPs towards PWD and its relationship to substance use (3, 34, 36, 37). PWD attributed these negative feelings to attitudes and beliefs held by HCPs related to having a disability. This finding has been shown extensively in earlier research. For example, Lewis and Stenfert-Kroese found that staff reported negative attitudes and emotions in caring for PWD, impacting the quality of care received (32). It is noteworthy that our study identified new insights into PWD being inaccurately labelled as substance users. Certain disability groups have been shown to suffer disproportionately from substance misuse (38). However, based on the findings by Chapman and Wu, the prevalence of illicit drug use, particularly in intellectual disabilities, is low (39). Some PWD may display challenging behaviours in the ED, especially in environments not conducive to their needs. This, coupled with lack of experience and sufficient training, might lead HCPs to attribute their behaviour to substance use. More research is warranted into the negative attitudes of HCPs towards PWD and its relationship to substance use.

PWD reported that ED care could be most improved if HCPs had a better understanding of the patient's identity, culture and situation. Accompanying micronarratives suggested that HCPs lack the knowledge and skills to competently treat the unique health concerns of PWD. Previous literature suggests that healthcare providers are aware that PWD are often treated unfairly by the healthcare system (40). Despite this, HCPs are reported to make assumptions about PWD's quality of life, values, and preferences, thus limiting their health care options and compromising quality of care (40). Examples include failure to provide pap smears or contraception options to women with disabilities due to the incorrect assumption that they were not sexually active (41). Furthermore, PWD are sometimes not screened for physical, sexual, or emotional abuse (42). These examples highlight the need for training on disability cultural competence for HCPs and it is essential that such curricula be developed in collaboration with PWD, their caregivers, and service providers.

PWD reported receiving good medical care in terms of diagnoses, testing, and symptom treatment, however this finding was not substantiated by the quantitative findings. This finding is in contrast with those of Iacono and Davis who found that PWD received delayed or inappropriate diagnostic procedures and that HCPs have failed to adequately understand the symptoms that PWD present during visits to the ED (36).

### Strengths and limitations

This study has noteworthy limitations. Firstly, despite efforts to collect micronarratives from a wide range of disability groups, the data are from a convenience sample and thus may not be representative of all PWD. Certain disability groups such as persons with a sensory disability (i.e., hearing loss/deafness) may have been under-represented in the sample given the small sample size. Secondly, the micronarratives collected are short compared to traditional qualitative interview-based research. As such, they may lack detail provided by in-depth interviews. Finally, interpretation of the shared ED experiences may have been restricted by the predetermined labels on the slider and triads. However, the thematic analysis of the micronarratives suggested that the variable choices were very relevant to many of the shared experiences among PWD. This concern is also mitigated given the pilot testing of the survey with service providers and clients with lived experience in advance of data collection to ensure relevancy.

The study has several strengths. To our knowledge, this is the first mixed-methods study to examine the ED care experiences among a heterogeneous group of PWD in Canada. Using a sensemaking approach provided new insights into the ED care experiences among PWD and empowered participants to interpret their own experiences, thus reducing researcher biases. Finally, within each slider and triad question all possible responses were either all negative, all positive, or all neutral such that no one response was perceived as being more ‘right’ than another, helping to mitigate social desirability bias.

### Recommendations

Based on the existing evidence and findings from this study, we feel that future quality improvement initiatives should focus on the following:

#### Incorporate a disability health curriculum into all aspects of Em training

A study conducted by Sapp et al. surveyed 237 EM residency training Program Directors in the United States to examine the curricula of disability health education in EM residency programs (9). The study suggested that incorporating disability health curriculum into all aspects of EM residency curriculum (i.e., lectures, journal clubs, research, and simulations) can improve confidence and skill in caring for PWD.

#### Design a curricula that emphasizes cultural humility

Cultural humility is skill related to lifelong learning that requires awareness, meaningful effort, respect, communication, and partnerships with the community (43). It requires the ability to recognize that culture is tied to power differences and societal inequalities. Although cultural competency is commonly discussed, a systematic review found that training in cultural competency might positively impact HCP knowledge but may not improve health outcomes (44). As such, curricula should emphasize a shift towards cultural humility training in medical school education and emergency medicine (EM) residency training (45, 46). *Design a curricula that emphasizes cultural humility.* Cultural humility is skill related to lifelong learning that requires awareness, meaningful effort, respect, communication, and partnerships with the community (43). It requires the ability to recognize that culture is tied to power differences and societal inequalities. Although cultural competency is commonly discussed, a systematic review found that training in cultural competency might positively impact HCP knowledge but may not improve health outcomes (44). As such, curricula should emphasize a shift towards cultural humility training in medical school education and emergency medicine (EM) residency training (45, 46).

#### Create community-based placements for medical students and/or residents to work with PWD

Research has shown that including individuals with lived experiences into training on caring for PWD had a positive effect on the self-reported comfort levels of medical students (47). Additionally, learning from a PWD may challenge the stigma related to disability and can provide new insights for HCPs without disabilities (46, 48).

#### Create community-based placements for medical students and/or residents to work with PWD

Research has shown that including individuals with lived experiences into training on caring for PWD had a positive effect on the self-reported comfort levels of medical students (47). Additionally, learning from a PWD may challenge the stigma related to disability and can provide new insights for HCPs without disabilities (46, 48).

## Conclusion

This study examined the ED care experiences among PWD at a single centre in Ontario, Canada. Our findings suggest that PWD were given too little attention to their needs, that it was more important for them to be treated with kindness/respect than to receive the best possible medical care, they felt powerless, judged, and ignored when visiting the ED, and require improved understanding of personal situation, identity, and culture as well as better communication between HCPs. Thematic analysis helped to further contextualize the findings and how best to improve the ED care experiences among PWD. Implementation of disability-health related curriculums in all health care provider curriculum/training as well as training in cultural humility should be considered and inform future implementation research.

## Data Availability

The raw data supporting the conclusions of this article will be made available by the authors, without undue reservation.

## Appendix 1

**Table:** Dyad and triad questions with possible answers.

Question	Possible answer
Micro-narrative prompts	
Share an example of how visiting the emergency room helped or harmed you or someone you were at the hospital with.	Micro-narrative recorded by participant
Tell a story about the best or worst experience you or someone you were with had in the emergency room.	Micro-narrative recorded by participant
Give an example of an experience that went very well or very badly for you or someone you were with at the emergency room.	Micro-narrative recorded by participant
Dyads	
During the emergency room visit, the patient's personal situation, identity or culture received…	1) Far too little attention; 2) Far too much attention; 3) This question does not relate to the story I shared/I do not want to answer
The events in the story were mostly about…	1) How the hospital works; 2) Emergency room staff; 3) This question does not relate to the story I shared/I do not want to answer
Based on the story shared, the patient's ability to pay for care or other costs (i.e., medicines, travel for care) received…	1) Far too little attention; 2) Far too much attention; 3) This question does not relate to the story I shared/I do not want to answer
Based on the story shared, the patient was given…	1) Too little attention to their needs; 2) Too much attention to their needs; 3) This question does not relate to the story I shared/I do not want to answer
During the visit, how much control/say did the patient have in making decisions about their care…	1) Too much control; 2) Too little control; 3) This question does not relate to the story I shared/I do not want to answer
In the experience shared, it was more important for the patient to…:	1) Receive the best possible medical care; 2) Be treated with kindness and respect; 3) This question does not relate to the story I shared/I do not want to answer
Triads	
During the events in the shared story, the patient was…	1) Judged; 2) Powerless/Not in control; 3) Ignored; 4) This question does not relate to the story I shared/I do not want to answer
During this story, the patient was…	1) Informed; 2) Empowered/In control; 3) Accepted/Valued; 4) This question does not relate to the story I shared/I do not want to answer
In the shared experience, the doctors, nurses or other emergency room staff…	1) Understood the situation; 2) Shared important information; 3) Showed they cared; 4) This question does not relate to the story I shared/I do not want to answer
The patient's shared experience was affected most by:	1) Wait times; 2) The medical care/testing provided; 3) How emergency room staff behaved towards the patient; 4) This question does not relate to the story I shared/I do not want to answer
After the patient left the emergency room, he/she was:	1) Not sure of what to do next; 2) Unclear about their health condition; 3) Unsupported in coping with their health concern; 4) This question does not relate to the story I shared/I do not want to answer
Based on the shared story, the following would most improve future emergency room care:	1) Better understanding of their personal situation, identity and culture; 2) Easier access to medical care; 3) Better communication between health care workers; 4) This question does not relate to the story I shared/I do not want to answer

## Appendix 2

**Table:** Focus group discussion questions.

Question	Study finding
Does this finding align with the experiences of your community? Why or why not? What other feelings come to mind when you think about ED care experiences for individuals who have a disability? Why might individuals with disabilities have more negative experiences than those who do not have a disability?	Negative feelings
Is this finding surprising to you? Why or why not? What does good communication look like to you? What do you think ED staff can do to improve communication with individuals who have a disability?	Poor communication between ED and staff
Does this finding surprise you? Why or Why not? Do you believe that the Kingston Health Sciences Centre ED staff is knowledgeable when it comes to treating individuals who have a disability?	Positive medical care received
What do you think the ED staff are doing well when it comes to treating individuals who have a disability? What is it about the ED care experience that might define it as being a positive one rather than negative?	Compassionate care
